# Ih modulates theta rhythm and synchrony in computer model of CA3

**DOI:** 10.1186/1471-2202-13-S1-P80

**Published:** 2012-07-16

**Authors:** Markus M Hilscher, Thiago Moulin, Yosef Skolnick, William W Lytton, Samuel A Neymotin

**Affiliations:** 1 Institute for Analysis and Scientific Computing, Vienna University of Technology, Vienna, Austria; 2 Neurodynamics Lab, Department of Neuroscience, Uppsala University, Uppsala, Sweden; 3 Medical Biochemistry Institute, Federal University of Rio de Janeiro, Rio de Janeiro, Brazil; 4 Neurosimulation Lab, SUNY Downstate Medical Center, Brooklyn, NY 11203, USA; 5 Kings County Hospital, Brooklyn, NY 11203, USA; 6 CUNY Brooklyn College, Computer Science Department, Brooklyn, NY 11210, USA

## 

Although the contributions of different hippocampal cell classes to oscillatory activity has been studied, the intrinsic membrane mechanisms contributing to the oscillations and neuronal synchronization are not well understood. The hyperpolarization-activated cation (HCN) channel is a voltage-gated ion channel potentially implicated in epilepsy. Ih, the current produced by HCN channels, plays an important role in regulating neuronal excitability, particularly in hippocampal and neocortical pyramidal neurons. Changes in HCN conductance have been associated with epilepsy, manifesting as discharges with aberrant synchrony [1]. In this study we investigated the role of Ih in pacing network oscillations and synchronizing activity. We studied the effects of modulating both the Ih time-constant and the Ih conductance level in a biophysical computer model of the CA3 region of the hippocampus using the NEURON simulator. Some of the manipulations could be realized *in-vitro* with pharmacological manipulation via the Ih blocker, ZD7288.

Our network consisted of 800 five-compartment pyramidal cells, 200 one-compartment basket cell interneurons, and 200 one-compartment oriens lacunosum-moleculare (O-LM) interneurons. All cells contained leak current, transient sodium current and delayed rectifier current. Additionally, pyramidal cells contained potassium type A current and pyramidal and OLM cells had Ih current. Cell classes were interconnected probabilistically with AMPA/NMDA, and two classes of GABAA synapses. The O-LM cells formed synapses on the distal dendrites of pyramidal cells, while the basket cells synapsed proximally on pyramidal and other basket cells. Pyramidal cells synapsed on both types of interneurons with AMPA/NMDA synapses. All synapses were bombarded with external Poisson inputs to generate network activity. We used Kendall’s tau correlation to measure the synchrony between pairs of pyramidal cells and performed FFT analysis on local field potentials generated by the pyramidal cells to measure rhythmic activity.

At baseline, OLM cells fired preferentially at the theta frequency, causing periodic inhibition/disinhibition of pyramidal cells [2]. Although lowering the Ih conductance of pyramidal cell distal dendrites did not change average firing rates of pyramidal cells, the delay to pyramidal cell synchronization increased. Delayed synchronization was associated with a delay in the emergence of pyramidal interneuron network gamma (PING; in PING, pyramidal cells drive basket cells via AMPA/NMDA receptors and basket cells in turn inhibit the pyramidal cells through GABAergic synapses). This mechanism depends on stabilization via pyramidal cell synchronization. Analysis of the simulated local field potential spectral power showed that Ih conductance level correlated with the peak theta rhythm, from ~6.5 - 8.5 Hz.

Our model demonstrates that changes in conductance of HCN channels can modulate hippocampal network rhythms and synchrony. These effects could be tested *in-vivo* and *in-vitro*, under neuromodulatory or pharmacological control. Our model also predicts that hippocampal networks may become more prone towards epilepsy with alterations in the level of HCN channel expression.
